# The Cost of Pushing Too Hard: Academic Self-Efficacy and Self-Alienation Link Achievement-Oriented Parental Psychological Control to Disruptive Behaviors

**DOI:** 10.3390/bs16091493

**Published:** 2026-08-26

**Authors:** Lixia Wang, Zekun Zhang, Xiaobo Xu, Weiguo Pang

**Affiliations:** 1 Department of Higher and Vocational Education, Shanghai Teacher Institute, Shanghai 200233, China; wanglixia@sti.edu.cn; 2 School of Psychology and Cognitive Science, East China Normal University, Shanghai 200062, China; zhangzk@stu.ecnu.edu.cn; 3 School of Psychology, Shanghai Normal University, Shanghai 200234, China

**Keywords:** achievement-oriented parental psychological control, academic self-efficacy, self-alienation, disruptive behavior, chain mediation

## Abstract

Although achievement-oriented parental psychological control (APPC) has been associated with several forms of adolescent maladjustment, little is known about its relationship to disruptive behavior at school. Drawing on self-determination and social cognitive theories, this study tested whether academic self-efficacy and self-alienation mediated this association. A sample of 544 middle school students in grades 7 and 8 (grade 7: *n* = 341, 62.68%; grade 8: *n* = 203, 37.32%; 48.35% female) across 20 classrooms at a public school in a mid-sized city in central China completed self-reported measures of APPC, academic self-efficacy, self-alienation, and disruptive behavior. Path analysis revealed that APPC was indirectly associated with disruptive behaviors via academic self-efficacy and self-alienation. The findings highlight the importance of self-perception as a key mechanism linking APPC to adolescent behavioral problems, offering valuable insights for future interventions.

## 1. Introduction

Often linked to family honor and children’s prospects, academic achievement carries considerable weight in Chinese families (Lin et al., 2024). Therefore, some parents make approval contingent on performance, withdraw affection after poor results, or compare their child with higher-achieving peers, which can be labeled as achievement-oriented parental psychological control (APPC; Soenens et al., 2010).

Research has linked APPC to depression, perfectionism, fear of failure, reduced curiosity, poorer academic performance, and lower life satisfaction in adolescents (Bleys et al., 2018; Cacioppo et al., 2013; Goldner & Scharf, 2025; Won et al., 2026; Wuyts et al., 2015). Disruptive classroom behaviors (e.g., talking out of turn, being out of one’s seat, and being off task) are also associated with diverse negative outcomes, including poorer academic achievement, increased psychological distress, and impaired interpersonal relationships (Marshall et al., 2024). Thus far, the link between APPC and disruptive behaviors remains insufficiently understood. Given their negative influence on adolescents’ adaptive development, it is theoretically and practically important to examine the relationship between these two constructs, elucidate the underlying mechanisms, and propose evidence-based strategies for potential intervention programs. Accordingly, drawing on self-determination theory (SDT; Deci & Ryan, 2000) and social cognitive theory (SCT; Bandura, 1997), the present study examined whether adolescents’ academic self-efficacy and self-alienation function as mediators bridging APPC and heightened disruptive behaviors.

## 2. APPC and Disruptive Behavior

In diverse samples, empirical evidence has consistently confirmed associations between parental psychological control and a range of externalizing problems across adolescence (Brenning et al., 2019; Romm & DiLissio, 2025). Extending this relationship to the educational context, in their longitudinal study, Xu et al. (2020) demonstrated that parental psychological control predicted students’ academic maladjustment in the classroom (e.g., disruptive behaviors, cheating, self-handicapping) one year later. As a domain-specific manifestation of general parental psychological control, APPC shares core conceptual features such as guilt induction, love withdrawal, and authority intrusion (Soenens et al., 2010). This conceptual overlap suggests that APPC may be positively associated with disruptive behaviors. Furthermore, as indirect evidence, recent research has demonstrated negative associations between APPC and emotion regulation (Safdar & Khan, 2019) and self-control (Bai et al., 2020), both of which are well-established proximal predictors of disruptive behaviors (Duncombe et al., 2013). Collectively, these theoretical and empirical insights suggest that APPC may be positively associated with disruptive behaviors.

### 2.1. Academic Self-Efficacy as a Mediator

Academic self-efficacy refers to a student’s belief in their ability to master learning tasks and achieve academic goals (Zimmerman, 2000). According to SCT, self-efficacy involves a core process through which the social environment shapes behavior (Bandura, 1997). By making parental recognition contingent upon academic performance, APPC communicates to the child that their intrinsic worth is conditional and that academic failure carries severe relational consequences (Ching et al., 2021). Consequently, students may focus less on developing competence and more on avoiding negative evaluations, which could increase performance anxiety and reduce mastery experiences from which self-efficacy develops (García-Pérez et al., 2019; Goldner & Scharf, 2025).

Further, academic self-efficacy shapes how students regulate their learning behaviors. Students with high academic efficacy beliefs usually display stronger learning motivation, use more effective strategies to tackle learning challenges, and recover quickly from learning setbacks; by contrast, students who doubt their academic ability are more likely to experience frustration from learning activities and view the classroom as a stressful environment (Schunk & DiBenedetto, 2022). The frustration–aggression hypothesis posits that feelings of frustration can breed aggression (Berkowitz, 1989), indicating that less efficacious students are more likely to express academically related frustration as a disruptive behavior in the classroom (Rocchino et al., 2017). Accordingly, we propose that academic self-efficacy may mediate the link between APPC and students’ disruptive behaviors.

### 2.2. Self-Alienation as a Mediator

Self-alienation refers to feeling disconnected from one’s emotions, values, and identity (Wood et al., 2008). From an SDT perspective, self-alienation can manifest when people internalize the standards imposed by others and act to avoid disapproval rather than from personal endorsement (Weinstein et al., 2012). APPC may create these conditions by pressuring adolescents to pursue externally defined achievement goals. If parental approval repeatedly depends on results, adolescents may learn to distrust or suppress their own academic interests and emotional reactions. Over time, they may come to feel that their authentic self is unacceptable or irrelevant, which is central to self-alienation (Wood et al., 2008).

Conceptually, authentic self-awareness serves as a core psychological resource for autonomous motivation and self-directed volition (Kernis & Goldman, 2006). When students are entrenched in self-alienation, they may lack clarity regarding their core values and are frequently unable to establish meaningful and autonomous academic goals (Kim et al., 2018). Additionally, chronic self-alienation may render individuals more vulnerable to the depletion of cognitive and emotional regulatory resources, increasing the likelihood of diminished self-control (Peng et al., 2026). Therefore, when faced with academic challenges or classroom boredom, self-alienated students are more likely to experience behavioral disengagement and fail to inhibit impulsive urges, ultimately manifesting as more frequent disruptive behaviors in the classroom. Overall, a reasonable proposition is that self-alienation may mediate the relationship between APPC and disruptive behaviors.

### 2.3. Chain Mediation Pathway: From Academic Self-Efficacy to Self-Alienation

According to SDT, by frustrating basic psychological needs, reduced academic self-efficacy could be associated with self-alienation. Consistent with this idea, Thomaes et al. (2017) revealed that when individuals experience frustration related to competence needs, they report heightened feelings of self-alienation and diminished authenticity. According to the hierarchical model of self-concept, domain-specific self-cognition may feed into broader self-representations (Geurten et al., 2018; Marsh, 2008). When students repeatedly doubt their academic capabilities, this negative self-cognition may generalize to their broader identity, instigating a sense of aimlessness and self-uncertainty (Zhang, 2026). Taken together, these accounts support a pathway from reduced academic self-efficacy to greater self-alienation.

### 2.4. Present Study

Integrating the above theoretical arguments, we propose that the lowered academic self-efficacy associated with APPC may be linked to heightened experiences of self-alienation among adolescents, which in turn may be accompanied by elevated levels of disruptive behaviors. This cascade is consistent with SDT and the hierarchical model of self-concept, both of which highlight that decreased academic self-efficacy may generalize to a broader sense of self-alienation. Specifically, this study tests three hypotheses: (1) APPC is negatively associated with academic self-efficacy, which in turn relates to more disruptive behaviors (Hypothesis 1); (2) APPC is positively associated with self-alienation, which in turn relates to more disruptive behaviors (Hypothesis 2); and (3) APPC is associated with elevated disruptive behaviors via a sequential indirect pathway through diminished academic self-efficacy and heightened self-alienation (Hypothesis 3).

## 3. Method

### 3.1. Participants and Procedure

Using convenience sampling, 698 middle school students were initially recruited from a public school in a mid-sized city in central China. Students in Grades 7 and 8 (aged 11–15 years) across 20 classes were invited to participate voluntarily. Standard informed consent was obtained from all the students and their guardians, and the students were free to withdraw from the study at any time. Two directed-attention check items were randomly embedded in the survey (e.g., “This is an attention check item; please select ‘Strongly Agree’”) to safeguard data quality and eliminate careless or random responding among early adolescents. Following our primary screening protocol, participants who failed at least one attention check item were excluded (*n* = 154)^1^. The final sample comprised 544 participants (48.35% female). Of these, 341 students (62.68%) were in Grade 7 and 203 students (37.32%) were in Grade 8. This study was approved by the institutional review board (IRB) (Protocol No. 2023-065) of the corresponding author’s affiliated university. With local teachers’ support, trained research assistants conducted the in-class survey during self-study hours, and the students participated voluntarily.

### 3.2. Measures

APPC. This variable was measured using a 9-item subscale from the Dependency-oriented and Achievement-oriented Psychological Control Scale (Soenens et al., 2010), previously validated in a Chinese adolescent sample and demonstrating good psychometric properties (Deng et al., 2019). A sample item is, “My father/mother only shows his/her love for me if I get good grades.” Participants rated their fathers and mothers separately on a 5-point Likert scale from 1 (strongly disagree) to 5 (strongly agree), with higher scores indicating greater APPC. To investigate potential parent-specific differences, we analyzed paternal and maternal scores separately. Given their strong bivariate correlation (*r* = 0.65, *p* < 0.001) and to mitigate potential multicollinearity while capturing cumulative family-level psychological control, we aggregated both ratings into an overall APPC score. Subsequent sensitivity analyses confirmed that separate paternal, maternal, and combined APPC models yielded consistent correlation and mediation patterns, demonstrating empirical stability across parental targets. The scale demonstrated good internal consistency in the present sample, with Cronbach’s α values of 0.86, 0.88, and 0.92 for paternal, maternal, and overall combined APPC, respectively. Meanwhile, the McDonald’s ω values were 0.88, 0.91, and 0.93 for paternal, maternal, and overall combined APPC, respectively.

Academic Self-Efficacy (ASE). This variable was assessed using a five-item scale adapted from the Patterns of Adaptive Learning Scales (PALS; Midgley et al., 2000). The Chinese version of this scale has been validated in a Singaporean adolescent sample and has shown sound psychometric properties (Nie et al., 2011). A sample item is, “Even if the course material is difficult, I can master it.” The participants rated items on a 5-point Likert scale ranging from 1 (strongly disagree) to 5 (strongly agree), with higher scores reflecting greater academic self-efficacy. In the present sample, the scale demonstrated acceptable internal consistency (Cronbach’s α = 0.78, McDonald’s ω = 0.79).

Self-Alienation (SA). This variable was assessed using four items from the Authenticity Scale (Wood et al., 2008), previously validated in a Chinese adolescent sample and demonstrating good psychometric properties (Song et al., 2020). An example item is, “I do not know how I really feel inside.” The participants rated it on a 7-point Likert scale ranging from 1 (does not describe me at all) to 7 (describes me very well), with higher scores indicating greater self-alienation. In the present sample, this scale showed good internal consistency, with Cronbach’s α = 0.84 and McDonald’s ω = 0.84.

Disruptive Behavior (DB). This variable was measured using five items adapted from the PALS (Midgley et al., 2000), previously validated in a Chinese adolescent sample and demonstrating good psychometric properties (Xu et al., 2020). A sample item is, “I sometimes get into trouble with my teacher during class.” The participants rated each item on a 5-point Likert scale from 1 (not at all true) to 5 (very true), with higher scores indicating greater disruptive behavior. In the current sample, the scale showed acceptable internal consistency, with Cronbach’s α = 0.73 and McDonald’s ω = 0.77.

Control Variables. Participants’ gender, grade level, and family socioeconomic status (SES) were included as covariates in all path analyses. Family SES was calculated as the mean of both parents’ highest educational attainment, rated on a 5-point scale. Paternal and maternal educational levels were strongly correlated (*r* = 0.59, *p* < 0.001). Missing values (three entries in total) were imputed using the median before averaging.

### 3.3. Data Analytic Strategy

Statistical analyses were conducted in consecutive steps using R (version 4.6.0; R Core Team, 2026) with the psych (Revelle, 2026) and lavaan (Rosseel, 2012) packages. First, descriptive statistics and bivariate Pearson correlations were computed to examine the preliminary associations among all the research variables. Second, potential common method bias was examined using a two-pronged diagnostic approach: Harman’s (1976) single-factor test via unrotated principal component analysis, and the common method variance (CMV) approach within a confirmatory factor analysis (CFA) framework using maximum likelihood (ML) estimation (Podsakoff et al., 2003). Finally, path analysis was conducted to evaluate the hypothesized chain-mediated model, controlling for gender, grade level, and SES. Indirect effects were evaluated using 5000 non-parametric percentile bootstrap resamples, with effects considered statistically significant if their 95% bias-corrected bootstrap confidence intervals excluded zero (Preacher & Hayes, 2008). Intraclass correlation coefficients (ICCs) were evaluated for all the primary variables to assess potential classroom-level clustering. The ICCs for APPC (0.0056), ASE (0.0232), SA (0.0020), and DB (0.0273) were all well below the 0.050 threshold for multilevel analysis (González-Romá & Hernández, 2017; LeBreton & Senter, 2008; Peugh, 2010), indicating that a classroom-level multilevel model was unnecessary.

## 4. Results

### 4.1. Common Method Bias Check

Harman’s single-factor test revealed the largest unrotated factor, accounting for 28.34% of the total variance, well below the conservative 40% threshold (Podsakoff et al., 2003). Moreover, a common latent factor (CLF) was added to the measurement model (χ^2^ = 1031.211, comparative fit index [CFI] = 0.929, Tucker–Lewis index [TLI] = 0.921, root mean square error of approximation [RMSEA] = 0.049, standardized root mean square residual [SRMR] = 0.051; with all standardized factor loadings ranging from 0.28 to 0.89, *p*s < 0.05) and linked to all observed indicators (Williams et al., 2010). Adding the CLF produced minimal practical improvement in model fit (∆CFI = 0.020, ∆TLI = 0.018) despite a significant chi-square difference (∆χ^2^ = 195.694, *p* < 0.001). Crucially, variance partitioning showed that the CLF accounted for only an average of 2.32% of the indicator variance, substantially below the 25.0% threshold established in empirical literature (Fuller et al., 2016; Podsakoff et al., 2012; Williams et al., 2010). Taken together, this evidence suggests that common method bias is unlikely to substantially confound these relationships, although statistical diagnostics alone cannot fully rule out potential single-source variance.

### 4.2. Descriptive Statistics and Intercorrelations

Table 1 presents the means, standard deviations, and bivariate correlations among the study variables. Maternal and paternal APPC were strongly and positively correlated (*r* = 0.65, *p* < 0.001), indicating high inter-parental convergence. APPC was positively correlated with self-alienation (*r* = 0.33, *p* < 0.001) and disruptive behavior (*r* = 0.29, *p* < 0.001), and negatively correlated with academic self-efficacy (*r* = −0.19, *p* < 0.001). Meanwhile, academic self-efficacy was negatively correlated with both self-alienation (*r* = −0.25, *p* < 0.001) and disruptive behavior (*r* = −0.19, *p* < 0.001). Further, self-alienation was positively correlated with disruptive behavior (*r* = 0.23, *p* < 0.001).

### 4.3. Mediation Analyses

**Figure 1:**
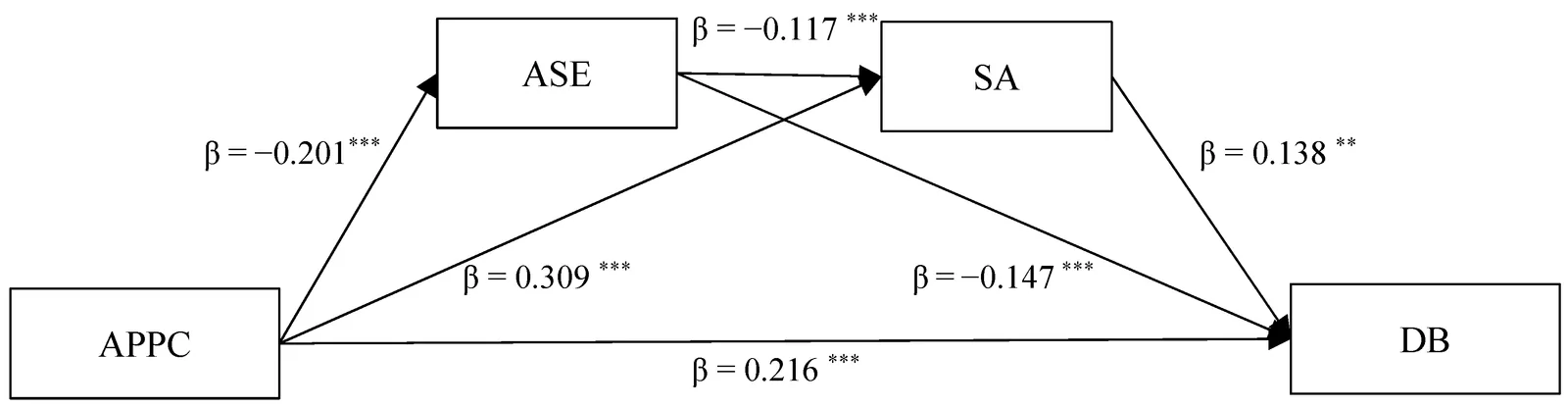
Structural Path Model with Standardized Coefficients. Note. APPC = achievement-oriented parental psychological control; ASE = academic self-efficacy; SA = self-alienation; DB = disruptive behavior. For visual clarity, the effects of demographic covariates (gender, grade level, and family SES) and residual variances are estimated in the path analysis but omitted from the graphical presentation. *** *p* < 0.001, ** *p* < 0.01.

The hypothesized chain mediation model was tested with APPC as the independent variable, academic self-efficacy and self-alienation as sequential mediators, and disruptive behavior as the outcome variable, with gender, grade, and SES (i.e., the average of both parents’ highest educational attainment) entered as covariates. Table 2 presents the bootstrapping results with 5000 resamples for all indirect pathways. Consistent with Hypothesis 1, APPC negatively predicted academic self-efficacy (β = −0.201, *p* < 0.001), which in turn negatively predicted disruptive behavior (β = −0.147, *p* < 0.001), yielding a significant indirect effect (β = 0.030, *p* = 0.008, 95% CI [0.010, 0.054]). Regarding Hypothesis 2, APPC positively predicted self-alienation (β = 0.309, *p* < 0.001), which in turn positively predicted disruptive behavior (β = 0.138, *p* = 0.001), resulting in a statistically significant indirect effect (β = 0.043, *p* = 0.003, 95% CI [0.015, 0.071]). Consistent with Hypothesis 3, the sequential mediation chain from APPC to disruptive behavior through diminished academic self-efficacy and heightened self-alienation was statistically significant (β = 0.005, *p* = 0.036, 95% CI [0.001, 0.010]), as illustrated in Figure 1. Overall, the total effect of APPC on disruptive behavior was significant (β = 0.292, *p* < 0.001, 95% CI [0.202, 0.383]).

To assess the robustness of our findings to potential measurement errors, we evaluated the structural mediation model using a latent variable structural equation modeling (SEM) framework with 5000 bias-corrected bootstrap re-samples. The latent structural model fit the data well (χ^2^/df = 2.171, CFI = 0.926, TLI = 0.918, RMSEA = 0.046, SRMR = 0.050). The latent SEM yielded virtually identical path estimates and confirmed the significance of the sequential mediation chain (β = 0.009, *p* = 0.052, 95% CI [0.002, 0.019]). Although the symmetric normal-theory Wald test yielded a marginal *p*-value due to the inherent skewness of the distribution in compound product estimators, the 95% bias-corrected bootstrap confidence interval strictly excluded zero, confirming statistical significance in accordance with established mediation guidelines (Hayes & Little, 2022; Preacher & Hayes, 2008) and supporting the stability of our observed composite findings.

## 5. Discussion

To the best of our knowledge, this is the first study to investigate the mediating mechanisms through which APPC is linked to disruptive behavior. Consistent with our theoretical framework, the findings revealed three significant indirect pathways: (1) APPC was indirectly associated with disruptive behavior via reduced academic self-efficacy; (2) APPC was indirectly associated with disruptive behavior via heightened self-alienation; and (3) academic self-efficacy and self-alienation served as a sequential indirect pathway linking APPC to disruptive behavior.

### 5.1. Academic Self-Efficacy Mediates the Link Between APPC and Disruptive Behavior

The indirect path from APPC to disruptive behavior through academic self-efficacy is theoretically consistent with the SCT account that environmental factors generally shape adolescents’ developmental outcomes through self-cognitive processes (Bandura et al., 1996). According to Soenens et al. (2010), APPC communicates an achievement-contingent worth to adolescents. Students who chronically receive such information may become more sensitive to failure cues and doubtful about their learning abilities (García-Pérez et al., 2019), corroborating prior work demonstrating that psychologically controlling parenting undermines children’s academic self-efficacy (Wang et al., 2023; Xu et al., 2017). Furthermore, consistent with the frustration–aggression hypothesis positing that unfulfilled needs can manifest as behavioral outbursts and interpersonal disruption (Berkowitz, 1989), this study revealed a negative link between academic self-efficacy and disruptive behaviors. Specifically, students with higher self-efficacy are more confident in tackling academic challenges, employ more effective learning strategies, and deal with learning setbacks more resiliently (Schunk & DiBenedetto, 2022). By contrast, students with low self-efficacy are more likely to withdraw from challenging tasks and tend to view the classroom as a stressful environment, which in turn increases the likelihood of their resorting to disruptive behaviors to express academically related frustration (Rocchino et al., 2017).

### 5.2. Self-Alienation Mediates the Link Between APPC and Disruptive Behavior

According to SDT, self-alienation may arise when individuals are forced to achieve externally imposed values and standards (Wood et al., 2008). Empirically, Li et al. (2026) identified a negative link between parental psychological control and authenticity, in which adolescents reported greater self-alienation when they experienced, recalled, or imagined higher levels of parental psychological control. Extending this rationale to an achievement-oriented context, our findings suggest that by chronically imposing performance-contingent standards of self-worth, APPC creates a frustrating environment associated with adolescents’ feelings of disconnection from their true selves (Assor et al., 2004). Furthermore, the positive correlation between self-alienation and disruptive behavior indicates a modest but meaningful association: Adolescents who feel alienated from themselves are more likely to engage in rule-breaking behaviors within the classroom. This pattern is consistent with prior evidence showing that alienation is positively associated with aggressive and disruptive behaviors (e.g., Piñuela et al., 2023). As mentioned above, alienated students typically lack clear learning goals (Kim et al., 2018), exhibit weaker self-control (Peng et al., 2026), and feel detached from their ongoing learning experiences (Kernis & Goldman, 2006). Consequently, they may be more prone to experiencing frustration and demonstrating disruptive behaviors.

### 5.3. Chain Mediation Pathway from Academic Self-Efficacy to Self-Alienation

Consistent with Hypothesis 3, our results revealed a sequential indirect pathway through academic self-efficacy and self-alienation linking APPC to adolescents’ disruptive behaviors. These results have several theoretical implications. First, they extend prior empirical work examining academic self-efficacy vis-à-vis achievement, engagement, and well-being (e.g., Kristensen et al., 2023; Schunk & DiBenedetto, 2022), with the current study directly testing its association with self-alienation as a distinct psychological outcome. The corresponding finding aligns with SDT, which highlights the importance of basic psychological need satisfaction in nurturing feelings of authenticity (Kernis & Goldman, 2006); accordingly, recent empirical studies have also confirmed a positive relationship between frustration related to competence needs and feelings of self-alienation (e.g., Thomaes et al., 2017). Further, according to the hierarchical model of self-concept, domain-specific self-evaluations can shape individuals’ global self-knowledge (Geurten et al., 2018; Marsh, 2008). If students lack confidence in their academic competence, this negative self-cognition may generalize to their broader identity and be associated with greater self-alienation (Zhang, 2026).

### 5.4. Practical Implications

The present findings offer several practical implications for intervention programs targeting adolescents’ disruptive behaviors. For parents, programs could be designed to remind them of the detrimental effects of APPC and encourage mastery-supportive strategies rather than performance-contingent practices to foster their children’s academic achievement (Joussemet et al., 2008). At the classroom level, teachers could consider strategies to scaffold students’ academic efficacy, such as designing challenging tasks appropriately, setting incremental goals, and providing process-oriented feedback (Hattie & Timperley, 2007). For students, interventions could help adolescents recognize self-alienation and equip them with effective coping strategies to buffer against stress from parental academic expectations (e.g., Kipfelsberger et al., 2022).

### 5.5. Limitations and Future Directions

This study has several limitations. First, as cross-sectional mediation cannot establish temporal order or causal mechanisms (Maxwell & Cole, 2007), bidirectional associations may exist between the research variables (e.g., disruptive behaviors may increase parental control). To address this limitation, we encourage future research employing longitudinal or experimental designs to examine the causal relationships between these variables. Second, although common method bias was not a major statistical concern in this study, the shared response tendency (i.e., subjective self-report) may have inflated associations. Therefore, future studies would benefit from multi-informant measures for data collection (e.g., teacher- or peer-rated disruptive behaviors). Third, as APPC still demonstrated a significant direct association with disruptive behaviors after controlling for the current mediators, future research could explore additional pathways or unmeasured confounding factors, such as emotion regulation and self-control (Bai et al., 2020; Beliveau et al., 2023; Soares et al., 2026). Fourth, it remains unclear under which boundary conditions APPC is less likely to be associated with disruptive behavior. To address this question, future studies could explore whether protective factors (e.g., peer acceptance, teacher support, and adolescent resilience) can interrupt the mediation cascade revealed in the current study. Finally, because the data were obtained from a single school, caution is needed when generalizing the results across different contexts. Although our sample size ensured adequate statistical power, future studies using multi-site sampling are required to verify the broader applicability of these findings.

## 6. Conclusions

The present study revealed a chain mediation model in which APPC was indirectly associated with disruptive behavior via academic self-efficacy and self-alienation, highlighting the importance of parenting practices and self-cognition in understanding disruptive behavior among adolescents.

## Figures and Tables

**Table 1:** Descriptive Statistics and Bivariate Correlations Among the Study Variables (*N* = 544).

Variable	*M*	*SD*	1	2	3	4	5	6
1. Paternal APPC	1.83	0.73	—					
2. Maternal APPC	1.71	0.73	0.65 ***	—				
3. Total APPC	1.77	0.66	0.91 ***	0.91 ***	—			
2. Academic Self-Efficacy	3.64	0.74	−0.20 ***	−0.14 ***	−0.19 ***	—		
3. Self-Alienation	3.27	1.50	0.30 ***	0.31 ***	0.33 ***	−0.25 ***	—	
4. Disruptive Behavior	1.66	0.58	0.27 ***	0.26 ***	0.29 ***	−0.19 ***	0.23 ***	—

Note. APPC = achievement-oriented parental psychological control. *** *p* < 0.001.

**Table 2:** Indirect Effects in the Chain Mediation Model (*N* = 544).

Pathway	B	β	*SE*	*p*	95% CI
APPC → ASE	−0.224	−0.201	0.045	<0.001	[−0.289, −0.111]
APPC → SA	0.699	0.309	0.041	<0.001	[0.225, 0.387]
ASE → SA	−0.359	−0.117	0.043	<0.001	[−0.261, −0.092]
ASE → DB	−0.116	−0.147	0.042	<0.001	[−0.229, −0.064]
SA → DB	0.053	0.138	0.042	0.001	[0.051, 0.219]
APPC → DB (Direct effect)	0.189	0.216	0.046	<0.001	[0.123, 0.305]
APPC → ASE → DB	0.026	0.030	0.011	0.008	[0.010, 0.054]
APPC → SA → DB	0.037	0.043	0.014	0.003	[0.015, 0.071]
APPC → ASE → SA → DB	0.004	0.005	0.002	0.036	[0.001, 0.010]
Total indirect effect	0.068	0.077	0.019	<0.001	[0.042, 0.115]
Total effect	0.256	0.292	0.046	<0.001	[0.202, 0.383]

Note. APPC = achievement-oriented parental psychological control; ASE = academic self-efficacy; SA = self-alienation; DB = disruptive behavior. B = unstandardized coefficient; β = standardized coefficient; *SE* = bootstrap standard error for standardized estimates. The covariates (gender, grade, and SES) were controlled in the model. The explained variances (R^2^) and residual variances for endogenous variables were as follows: ASE (R^2^ = 0.075), SA (R^2^ = 0.158), and DB (R^2^ = 0.184). Estimates and 95% percentile bootstrap confidence intervals were derived from 5000 non-parametric bootstrap resamples using a fixed random seed.

## Data Availability

A de-identified dataset can be found in the Supplementary Materials.

## Note

1 Independent-samples *t*-tests indicated systematic baseline differences between excluded (*n* = 154) and retained (*n* = 544) participants: excluded adolescents reported higher overall APPC (*t* = 4.736, *p* < 0.001, *d* = 0.522), higher SA (*t* = 3.135, *p* < 0.001, *d* = 0.302), more DB (*t* = 5.658, *p* < 0.001, *d* = 0.670), and lower ASE (*t* = −1.972, *p* < 0.001, *d* = −0.189). However, a sensitivity analysis re-estimating the sequential mediation model on the full recruited sample (*N* = 698) showed that the focal sequential indirect effect remained statistically robust and virtually unchanged (β = 0.005, 95% CI [0.001, 0.013]), confirming that the documented indirect paths were not artifacts of participant exclusion.

## Supplementary Materials

The following supporting information can be downloaded at: https://www.mdpi.com/article/10.3390/bs16091493/s1.
